# Supplementary material for: Sleeping at the switch
Source: eLife. 2021 Aug 27;10:e64337. doi: 10.7554/eLife.64337 (PMC8452310; doi:10.7554/eLife.64337)
Supplement: Supplementary file 1. [file elife-64337-supp1.docx]

**Sleeping at the Switch**

Maude Bouchard B.Sc.^1,2^, Jean-Marc Lina Ph.D.^1,3,4^ Pierre-Olivier Gaudreault Ph.D.^1^, Alexandre Lafrenière B.Sc.^1^, Jonathan Dubé M.Sc.^1,2^, Nadia Gosselin Ph.D.^1,2^, & Julie Carrier, Ph.D. ^1,2^

^1^Center for Advanced Research in Sleep Medicine, Hôpital du Sacré-Coeur de Montréal, Montreal, Canada;

^2^Department of Psychology, Université de Montréal, Montreal, Canada

^3^Department of Electrical Engineering, École de Technologie Supérieure, Montreal, Canada

^4^Centre de Recherches Mathématiques, Université de Montréal, Montreal, Canada

**SUPPLEMENTARY MATERIAL**

***Supplementary Table 1 Demographic and polysomnographic variables for young and older subjects***

|  | ***Young (YO)*** | ***Older (OL)*** | ***Main effect (p values)*** | | | ***Effect*** |
| --- | --- | --- | --- | --- | --- | --- |
|  | ***N = 30*** | ***N = 29*** | ***Age*** | ***Sex*** | ***Interaction*** |  |
| ***Demographic Variables*** |  |  |  |  |  |  |
| Sex (M/W) | 16/14 | 11/18 | N/A | N/A | N/A | -- |
| Education (year) | 15.4 ± 2.2 | 15.4 ± 3.4 | n.s. | n.s. | n.s. | -- |
| Polysomnographic Variables | |  |  |  |  |  |
| Sleep latency (min) | 8.2 ± 5.9 | 9.0 ± 7.3 | n.s. | n.s. | n.s. | -- |
| REM latency (min) | 97.7 ± 49.4 | 78.6 ± 29.2 | n.s. | n.s. | n.s. |  |
| Sleep duration (min) | 451.3 ± 36.0 | 408.9 ± 36.4 | **< 0.001** | n.s. | n.s. | **YO > OL** |
| Sleep efficiency (%) | 93.1 ± 5.6 | 85.6 ± 6.8 | **< 0.001** | n.s. | n.s. | **YO > OL** |
| Stage NREM (%) | 79.4 ± 5.0 | 81.3 ± 4.8 | n.s. | n.s. | n.s. | -- |
| Stage REM (%) | 20.6 ± 5.0 | 18.7 ± 4.8 | n.s. | n.s. | n.s. | -- |
| NREM stage N1 (%) | 7.6 ± 4.2 | 10.6 ± 4.5 | N/A | N/A | **< 0.01** | **(M) YO < OL**  **(W) n.s.** |
| NREM stage N2 (%) | 53.0 ± 6.1 | 60.0 ± 7.6 | **< 0.001** | n.s. | n.s. | **YO < OL** |
| NREM stage N3 (%) | 18.8 ± 5.9 | 10.7 ± 7.4 | N/A | N/A | **< 0.01** | **(M) YO > OL**  **(W) n.s.** |
| Min. of wake (C1) | 5.0 ± 17.9 | 4.8 ± 6.5 | n.s. | N/A | N/A | -- |

*Notes: Data expressed as mean ± SD. P values were considered significant at p < 0.05. M, men; ms, millisecond; N/A, non-applicable; NREM, Non-rapid eye movement sleep; C1, Cycle 1; n.s., non-significant; OL, older subjects; REM, Rapid eye movement sleep; W, women; YO, young subjects*
