# Supplementary material for: Sleeping at the switch
Source: eLife. 2021 Aug 27;10:e64337. doi: 10.7554/eLife.64337 (PMC8452310; doi:10.7554/eLife.64337)
Supplement: Transparent reporting form [file elife-64337-transrepform.pdf]

eLIFE
Westbrook Centre, Milton Road
Cambridge CB4 1YG
UK
P 01223 855340
W elifesciences.org
T @elife

eLife's transparent reporting form

We encourage authors to provide detailed information *within their submission* to facilitate the interpretation and replication of experiments. Authors can upload supporting documentation to indicate the use of appropriate reporting guidelines for health-related research (see EQUATOR Network), life science research (see the BioSharing Information Resource), or the ARRIVE guidelines for reporting work involving animal research. Where applicable, authors should refer to any relevant reporting standards documents in this form.

If you have any questions, please consult our Journal Policies and/or contact us: editorial@elifesciences.org.

Sample-size estimation

- You should state whether an appropriate sample size was computed when the study was being designed
- You should state the statistical method of sample size computation and any required assumptions
- If no explicit power analysis was used, you should describe how you decided what sample (replicate) size (number) to use

Please outline where this information can be found within the submission (e.g., sections or figure legends), or explain why this information doesn't apply to your submission:

We have selected the largest number of participants that were responding to the highly strict inclusion/exclusion criteria of the study and that we could include in the time available for testing. Each participant had to sleep two times at the laboratory.

Replicates

- You should report how often each experiment was performed
- You should include a definition of biological versus technical replication
- The data obtained should be provided and sufficient information should be provided to indicate the number of independent biological and/or technical replicates
- If you encountered any outliers, you should describe how these were handled
- Criteria for exclusion/inclusion of data should be clearly stated
- High-throughput sequence data should be uploaded before submission, with a private link for reviewers provided (these are available from both GEO and ArrayExpress)

Please outline where this information can be found within the submission (e.g., sections or figure legends), or explain why this information doesn't apply to your submission:

Criteria for exclusion/inclusion are described in the *Participants and protocol* section of the Material and Methods section.
Due to the strict selection criteria of the cohorts, we did not have any outliers.
Other questions : N/A

Statistical reporting

- Statistical analysis methods should be described and justified
- Raw data should be presented in figures whenever informative to do so (typically when N per group is less than 10)
- For each experiment, you should identify the statistical tests used, exact values of N, definitions of center, methods of multiple test correction, and dispersion and precision measures (e.g., mean, median, SD, SEM, confidence intervals; and, for the major substantive results, a measure of effect size (e.g., Pearson's r, Cohen's d)
- Report exact p-values wherever possible alongside the summary statistics and 95% confidence intervals. These should be reported for all key questions and not only when the p-value is less than 0.05.

Please outline where this information can be found within the submission (e.g., sections or figure legends), or explain why this information doesn't apply to your submission:

Slow waves and sleep spindles detection is described in the Material and Methods section under: "Slow waves detection" and "Sleep spindles detection".

The computing of the transition frequency is described in the Material and Methods section under: "Slow waves characteristics" while the computing of slow and fast switchers is described under: "Slow and fast switchers".

Our non-parametrical statistical analyses and resampling information are described in the: "Phase-Locked connectivity analyses" of the Material and Methods section.

for homeostatic decrase : section. For additional info and p : Figure legends.

When indicated, parametric tests (ANOVA) were performed. The parametric analyses are described in the "Slow and fast switchers modulation analysis" section of the Material and Methods section.

Additional statistical information (ex : p-value) can be found in the figures' legend.

(For large datasets, or papers with a very large number of statistical tests, you may upload a single table file with tests, Ns, etc., with reference to sections in the manuscript.)

Group allocation

- Indicate how samples were allocated into experimental groups (in the case of clinical studies, please specify allocation to treatment method); if randomization was used, please also state if restricted randomization was applied
- Indicate if masking was used during group allocation, data collection and/or data analysis

Please outline where this information can be found within the submission (e.g., sections or figure legends), or explain why this information doesn't apply to your submission:

Information about the age groups can be found in the *Participants and protocol* section.

Additional data files ("source data")

- We encourage you to upload relevant additional data files, such as numerical data that are represented as a graph in a figure, or as a summary table
- Where provided, these should be in the most useful format, and they can be uploaded as "Source data" files linked to a main figure or table
- Include model definition files including the full list of parameters used
- Include code used for data analysis (e.g., R, MatLab)
- Avoid stating that data files are "available upon request"

Please indicate the figures or tables for which source data files have been provided:

All codes and transformed data used for all the analyses and most specifically to produce all of the figures of the paper can be freely accessible using this link : https://github.com/jmlina/Slow_Wave_Switchers

This information and link was also added in a new section at the end of the paper under "Additional data files".

Dataset can not be shared as participants did not give consent for data sharing. For the raw data, a request needs to be formulated to the ethic committee of the Hôpital de Sacré-Coeur de Montréal, as raw data of human participants cannot be made public under Québec's law.

The data provided will be anonymized and some will be processed.
Researchers who request access to the data will need to provide their research protocol and their IRB approval for this protocol.

The documents will be studied by the owner of the database (Julie Carrier) who will then also submit to her institution's REB for authorization to share the data.
Data requests should be addressed to:
Julie Carrier (PI): julie.carrier.1@umontreal.ca
Sonia Frenette (in cc) : sonia.frenette@umontreal.ca
